# Clinic Atlanta Medical College

**Published:** 1876-01

**Authors:** 


### Clinic Atlanta Medical College.

SERVICE OF J. G. WESTMORELAND, M.D.

By CHARLES BOYD AND CHARLES NUTTING, Reporters.

Case I.—November 15, 1875.—J. E., a female æt. 36, unmarried; general appearance indicates no serious disease. There is cough with more or less muco-purulent expectoration pretty constantly, and aggravated by "taking cold." Also, a peculiar arrest of growth in the finger nails. Percussion and auscultation give no decided signs of pneumonic difficulty.

The case presented affords no symptom upon which clear and unmistakable diagnosis can be formed. The history just obtained from the patient shows that the present condition has existed for several years, and yet we have nothing but the general symptoms of cough and unnatural appearance of the nails from which to make out an opinion of the pathological condition. The first may be the result of various, and sometimes very slight causes, while the latter generally denotes the existence of long standing, serious disease of lungs or bronchia. In the absence of evidences, from physical signs, of tubercular deposit in the lungs, my conclusion is, that the lesion exists in the bronchia or lungs to a limited extent, and in a part which has escaped notice in the imperfect examination made. The apex, in which tubercle is most frequently found, gives resonance and tolerably perfect vesicular murmur, and yet a critical examination would probably give evidences of disease posterially toward the base of one or both lungs.

Under all the circumstances of this case, gentlemen, treatment is demanded, although no urgent, dangerous, symptoms are present. Doubtless, lurking permanent lesion exists in the respiratory organs, and although perhaps limited in extent and slow in progress, depressing influences, such as long exposure to cold, damp atmosphere, sudden change of temperature, poor diet, etc., are likely to develop dangerous and rapidly fatal symptoms at any time.

General treatment does not seem to be called for, further than the direction to take regularly nutritious and oily food in digestible quantities, and avoid sudden transition from heated to cold atmosphere without adequate protection.

Local applications should, I think, be made, and of such remedies as are likely to arrest the progress of chronic inflammation. We have many such remedies, but a very limited number that can be, with facility, carried to any extent within the air passages. Insufflation and injection have been proposed, as means of introducing catheretics or local alteratives into the respiratory tubes; but although any liquid preparation may be thus introduced, the danger from remedies used in this way is much more imminent than that from the ravages of the disease for which they may be given. Inhalation, the only practicable mode of applying remedies to all parts of the respiratory mucous membrane, cannot be practiced successfully with liquid nor solid forms of preparation. Powdered substances inhaled will rarely be carried further than the larynx and trachea, while the vapors from solutions of non-volatile substances convey the remedies in such dilute form that no decided impression can be made by them. Only such articles as can be made to assume the state of fumes can be introduced in any desirable strength. Resin, a stimulant of the mucous membranes particularly, and iodine, a valuable catheretic, are subject to this form of preparation, and may be taken into all parts of the air passages, diluted to any desirable degree of strength by atmospheric air.

A simple and economical inhaler is equal to the most expensive apparatus for this purpose. A small, salt-mouthed vial, warmed on a stove or in any other convenient way, will answer the purpose of an inhaler, and for the preparation of the fumes. One or two grains of iodine, placed in the vial thus heated, may be inhaled through the mouth or nostrils. This will be the prescription for the case before us, to be used at night or morning, every day, for a week, and after that time, once in two or three days. In order to prevent excessive coughing by the inhalation, an opiate, say one-fourth grain of morphine, may be taken an hour before the time of inhaling.

November 19. Has found some relief from cough since inhaling the iodine. The direction is to continue inhalation, but suspend the opiate.

November 22. No material change since last seen. Treatment continued.

Case II.—November 15. Alfred Cherry, colored, æt. twenty-five, looks feeble and emaciated, coughs frequently, expectorating mucus, has some difficulty in breathing, occasional pain in epigastrum, and voids more than double the usual amount of urine; has very little desire for food, and often suffers pain after eating.

The history and general symptoms of this case lead me to suspect renal difficulty as the foundation of his trouble. No positive opinion, however, can be formed in regard to his true condition until the urine has undergone careful examination. This will be attended to on his return, and further remarks in regard to it will be deferred for the present.

November 19. The specimen of urine brought by the patient is subjected to a test. First for albumen, by boiling, and finding none, litmus paper is subjected to its action without giving evidence of acid by reddening. The urinometer shows the usual specific gravity of healthy urine, say 1.15.

The results of this examination show the importance of thorough examination before settling the diagnosis upon which treatment is instituted. Had we relied upon the reasonable suspicion entertained of this case, when first presented, the treatment of abuminuria or diabetes would have been prosecuted without benefit. His feeble condition seemed to be the natural result of the renal disturbance, and all other symptoms were susceptible of explanation on this hypothesis. Since, however, unerring tests have proved its fallacy, we must push our investigation in an other direction. We have discovered a want of appetite for food, and evidences of imperfect digestion of that which is taken. Now, whether this be the prime difficulty in his case or not, evidently restoration of the digestive powers will be eminently beneficial. In order to this, it is necessary to decide whether this failure depends upon undue irritation or inflammation of the stomach, or a want of the normal excitement. No redness of the tongue is found, indicating the former, and we shall conclude that inactivity exists throughout the chylo-poietic viscera. There is probably not only a want of the usual muscular contraction of the stomach and bowels, but deficient secretion of gastric and pancreatic fluids. These secretions are necessary, the former to dissolve food in the stomach, the latter to emulsify or prepare oily food for absorption or appropriation.

To restore these functions should be our prime object. That which will give permanent vigor to the stomach and duodenum will probably also restore the proper amount of these digestive fluids, but in order to have food acted on in the stomach at once, we will endeavor to afford gastric juice artificially along with the tonic intended to restore it ultimately. Then, in connection with a gastric tonic, we shall administer pepsin, the active principle of gastric fluid, obtained from animals.

It will probably be sufficient in this case to afford artificial gastric juice, along with the gastric tonic, as follows:

℞.—Pepsin, . . . . . . . gr. lxxx.
Tinct. Gentian, . . . . . .
Water, . . . . . . . aa. fl.℥ij.
Mix.

S. Two teaspoonfuls at each meal, after shaking well.

November 22. Patient reports appetite improved, strength increased and urine very much less in quantity. Pepsin and gentian continued as before directed.

November 28. In general appearance Alfred shows decided improvement. He reports appetite good, no gastric uneasiness, urine about normal. The same prescription continued.

December 3. Patient looks sprightly and cheerful. Seeming to have entirely recovered, is discharged from the clinic.

Case III.—November 22. Levinia Wright has opacity of the cornea in one eye. Some swelling of the lids without much redness of the conjunctiva.

The redness and opacity of the cornea depend upon inflammation of that tunic. This is one of the structures in which inflammation does not produce redness. Even in diseased condition the vessels of the part do not seem to carry red globules. Corneitis is more dangerous to vision than inflammation of any external tunic of the eye, for the reason found in this case, opacity, by which entrance of light is permitted. Often this condition of the cornea remains after the inflammatory action subsides, and perpetual blindness is the result. In this case, gentlemen, we hope to restore transparency, should the effort to arrest the inflammation at once be successful. The disease, however, has existed for a week or more, and may have caused already deposits difficult of removal.

This disease, like ordinary conjunctivitis, requires great care in the application of local remedies. Disease in such delicate structures is often aggravated by the careless use of irritating substances. Even the acetate of lead, one of the mildest astringents, may be used injuriously in ophthalmia, by applying the imperfect solution of an impure salt, containing insoluble carbonate, oxide, etc., which act as irritants. Collyria often contain substances decidedly injurious to acute sore eyes, from their stimulating properties also. For these reasons local applications, taken all together, have not usually proved useful in acute ophthalmia.

In this case the astringent action of sulphate cadmium, of proper strength, will probably be serviceable. In order to soothe and protect, as well as constringe the inflamed, delicate tissue, it will be necessary to make the solution in some demulcent, and for this purpose the mucilage from the pith of sassafras is preferable, as follows:

℞.—Sulphate Cadmium, . . . . gr. ss.
Mucilage of Sassafras pith, . . . f℥j.

Dissolve.

Sig. Four or five drops in the eye twice a day.

In addition to the collyrium just prescribed, counter-irritation should be instituted at once, and vigorously. The means used for this purpose are, rubefacients, acupuncture, cups, leeches, blisters and pustulants. Of these, some serve the purpose of local depletion, and in the case before us, such are decidedly preferable. Cups, with scarification, to the temples and nape of the neck, will not only afford useful depletion in the neighborhood of the diseased part, but counter-irritation to some extent also. For adults this is preferable, but owing to the small size of this patient a blister, two by four inches, is directed to be applied across the back of the head and neck.

Case IV.—November 22. Prudence Chapman, colored, æt. twenty-four, suffered, some months ago, from rheumatism of the right shoulder, elbow and wrist. She now complains of stiffness and soreness of the right side of neck and shoulder.

In the first place, I would remark, that rheumatic inflammation is peculiar in its migratory character, manner of termination and results. Like ordinary inflammation, it causes pain, swelling, redness and fever, and usually attacks fibrous and muscular tissues. Hence the joints and muscles are generally involved. The neurilemma or nervous cords are also subject to attack, giving rise to what is some timescalled "nervous rheumatism." Unlike common inflammation, rheumatism rarely, if ever, terminates in suppuration, but frequently leaves deposits of semi-organized fibrin or other substance, interfering with the mobility of joints, and leading to occasional soreness and pain called "chronic rheumatism."

The question first to be decided is, upon what primary disturbance does this peculiar and strangely behaved inflammation depend? Does it depend upon the deposit, in the parts affected, of lithic acid or other substance found in excess in the blood, or does it arise from a peculiarly deranged condition of the nervous system? Both of these theories have been promulgated by pathologists, and, as students of medicine, your attention is invited to-day to reasoning on the subject. If the former view be correct, why is it that the inflammation is transferred in an hour from one joint to another? Certainly, if the morbific cause be deposited from the blood, in the part inflamed, it would not be transferred to another part so suddenly as rheumatism changes location. Indeed, it could not be changed at all.

The theory of nervous origin is certainly established by the sudden transition, which peculiarity is observed by many nervous affections, such as pleurodynia, and neuralgia of various parts. The best means of successful treatment also confirm this opinion of its pathology, from their known influence over the nervous centres. Quinine and opium, with blisters over the spinal column, evidently, to my mind, afford the best means of treatment. These influences are exerted solely upon the spinal nervous centre, —one as a counter-irritant and the other as a tonic,—and when rheumatism is thus relieved, we naturally conclude that the disease depends upon disturbance of this centre of the nervous system.

In this case the blister alone will probably be sufficient. I therefore direct that a blister, two by six inches, be applied over the cervical and dorsal spine.

November 26. Patient has relief from the disease, but suffers from soreness of the blister, which is irritable from improper dressing.

Case V.—November 26. Sallie Allen, æt. 20, is suffering with pain and tenderness at lower portion of abdomen, just inside the left hip. Menstruated last week, and since the cessation has suffered with the pain referred to.

This, gentlemen, is a specimen of a large class of cases that consult physicians on all occasions and in all seasons. The cause of trouble, doubtless, is engorgement of the ovary, which has existed since the conclusion of her last menstrual period. Difficulties of this kind are supposed to arise from postponed or suppressed menstruation, and it is not very generally understood why such state should exist after full and perfect menstruation. When this difficulty is found, under the circumstances of this case, it affords one of the evidences of intra-uterine irritability, or chronic inflammation, extending, probably, along the fallopian tubes. Now, this pain of the ovary will probably cease in a short time by the use of an opiate internally, and rubefacient action over the seat of trouble; but is liab'e to return at the next period, or at any intermediate time, in one or both ovaries, so long as the irritation of the womb exists.

In order to give relief, I inject one-sixth of a grain of sulph. morphia and one-sixtieth grain of sulphate of atropia in ten drops of water, and direct a sinapism on the left lower part of the abdomen.

This case, if such disease of the uterus, as we suspect, exists, should be transferred to the gynæcological clinic for treatment directed to the interior of the womb. This can be had by tent medicated with iodine, nit. silver, sulph. zinc, or other suitable remedy; or by injecting a solution of it into the cavity, in a manner and in quantity not to produce undue contraction of the organ.

---

### SERVICES OF PROF. W. F. WESTMORELAND, M.D.

By T. M. McINTOSH, M.D., Reporter.

*Diseased Testicle.*—This little boy, who is only four and a half years of age, has been brought to the clinic this morning to be treated for an enlarged testicle. The trouble, as we learn, commenced one year ago, and we have now a tumor the size of a goose egg or larger. Have we a disease of the testicle, or a disease of its membranes? That is, have we hydrocele or have we an organic disease of the testicle proper, or both? If it is hydrocele, we will be able to transmit light through the enlargement by making the skin tense and placing the tumor between ourselves and a window, or what is better, a lighted candle or lamp. If we find the tumor translucent, we know that it can be nothing but an effusion into the serous sack covering the testicle—a true hydrocele. Upon examination we have no transparency, so that it cannot be an ordinary hydrocele. We sometimes, however, have the tunica-vaginalis testis, filled with an opaque fluid, with blood, as in hematacele; or in some cases of hydrocele, we have the fluid colored with the red corpuscles of the blood, and in such cases there will be no transparency. In some portions of this tumor there is certainly fluid, as the fluctuation can be distinctly felt; at other points it cannot be detected. In such cases the only mode of determining, with certainty, the character of the trouble is by inserting an exploring trocar, which we will now do. You see that there has passed out only a small quantity of fluid, not enough to perceptibly reduce its size. The remaining mass is, to a greater extent at least, enlarged testicle, and from its rapid growth we would pronounce it malignant or encephaloid cancer. An extirpation of the testicle is the only treatment that promises favorable results; and as the parents have already given their consent, and the boy is in good condition, we will perform the operation at once.

The little patient was now placed under the influence of ether, and the testicle removed with the loss of very little blood, as the spermatic artery was ligated before the cord was severed. The lips of the wound were now brought together with silver sutures, and strips of adhesive plaster used to sustain and support the parts. In two or three weeks the patient returned with the parts healed and was dismissed cured. An examination of the testicle removed proved it to be an encephaloid cancer.

*Tertiary Syphilis.*—This colored man presents himself this morning in a pitiable condition. We have here syphilitic caries of the sternum, and of both clavicles. In addition, as you see, he has abscesses of the cervical glands of the neck, and an immense abscess in the axilla of right side. His general health, as is evident from his appearance, is greatly impaired—tells us that he suffers more or less pain in his extremities, which he attributes to rheumatism, but which is evidently syphilitic. It is impossible, as you have seen, to get anything like a correct history of his trouble, as he most positively denies ever having had syphilis in any form. That the disease of the bone, however, and other prominent symptoms present, are syphilitic there can be no doubt, and we shall treat him accordingly. The remedies that we prescribed for the woman at our last clinic, who had secondary syphilis, and in which mercury was the basis, are not admissible in this case, as mercury, in any form, would most likely be injurious in his present condition. While mercury is our favorite remedy in secondary syphilis, we rarely give it in the tertiary form of the disease. And while the iodide of potassium is the basis of treatment in almost every case of tertiary syphilis, we very rarely give it in the secondary form of the disease. The iodide of potassium in large doses is the only remedy that will rapidly improve the condition of this poor man. We will then freely open the abscesses that are not already discharging, and extend the openings in those that are not well drained, and inject all with the tincture of iodine for the purpose of stimulating the suppurating tissue. For his cure, however, we shall rely upon the following:

℞.—Iodide of potassium, - - - - - ℥ii.
Tincture of gentian, - - - - - ℥iiss.
Simple syrup, - - - - - - ℥iiss.
Water, - - - - - - - - ℥ii.

M. Sol. He will take a tablespoonful morning, noon, and night, in half pint of water.

It will be seen that the patient will take sixty grains at a dose and one hundred and eighty grains in the twenty-four hours, but each dose is diluted with half pint of water. Many physicians have assured me that their patients would not bear the potash in such large doses; that it so affected the stomach that they have been forced to withdraw the remedy. Upon inquiry, I have almost invariably found that they had failed to give it in a sufficient quantity of water to properly dilute it. If not properly diluted, it almost invariably produces an irritable condition of the stomach, causing indigestion, with more or less nausea and vomiting. If it be given properly diluted with gentian in some form, it increases the appetite and digestion. I am yet to see the first case of tertiary syphilis in which I have not been able to give the iodide of potassium in from forty to sixty grains at a dose. I have had one case where the salt produced a terrible coryza with irritation or inflammation of the mucous membrane of the fauces and larynx with great distress, but by commencing with small doses, and persisting in its use, I finally overcame the trouble, and the sufferer was able to take the remedy. At the end of a week the patient returned, much improved in every particular.

*Stricture of the Urethra.*—This man presents himself to-day to be treated for stricture of the urethra, from which, he tells us, he has suffered more or less for twelve years. Upon examination we find two strictures, one anterior to the curve or bulb, and one at the bulb or curve, and extending to the membranous portion of the urethra. At the strictures, particularly the one nearest the bladder, the urethra is exceedingly contracted, so small that it was with great difficulty, as you saw, to introduce the smallest filiform bougie, and after its introduction could not introduce the divulsor over the bougie, and through the stricture, until the bougie had remained for some time in the urethra. There are several modes of treating stricture, and each plan or mode is applicable in a certain class of cases. No plan is applicable in all. There are a certain number of cases that we may cure by simply dilating with bougies, but all are not curable by this plan. In a certain number the best plan is a section of the stricture from within, or internal urethrotomy, as it is called. In other cases, again, the best, and in some the only mode that promises success, is a section of the stricture from without, or external urethrotomy or perineal section, if the section is made in the perineum. Another mode, and one which for past few years has been very popular, particularly in certain localities, is the rapid rupture or tearing the strictured portion of the urethra with a divulsor. The instrument that I hold in my hand is Sir Henry Thompson's divulsor, and, as you see, consists of two blades, and when closed and ready to introduce into the bladder, resembles, and is about the size of, a number five metallic bougie. By turning a screw at the end you see the blades are separated. It is by separating the blades, after the instrument has been introduced, that the stricture is ruptured and dilated to the extent of receiving the largest bougie. At some future time we will discuss internal and external urethrotomy, and the class of cases in which they are more particularly applicable. To-day we shall treat this case with the divulsor, by *divulsion*, as it is called. In this instrument we have Gouley's improvement, which consists in the small opening in the end, and the tunnel on the side, the object being to pass it over a filiform bougie, the bougie acting as a guide to the divulsor.

The instrument was now passed over the filiform into the bladder, and after adjusting it so that the largest part, when dilated, would correspond with the strictured portions of the urethra, the screw was rapidly turned and the blades separated to their fullest extent. The screw was now turned in the opposite direction, the blades closed, the instrument removed, and the largest size metallic bougie introduced without the least trouble. All the treatment necessary after such rupture or *divulsion* is the daily introduction of the bougie for three or four weeks, and later once every two or three weeks.

### EYE AND EAR CLINIC.

PROFESSOR A. W. CALHOUN., M.D.

(*Continued from page 474 of November Journal.*)

CASE XXVI.—*Scrofulous Keratitis*, (*Inflammation of the Cornea.*) S. K., colored boy, æt. sixteen, Atlanta. There is a scrofulous appearance about the patient, that would lead most of you at once to the conclusion that his disease belonged to that class. Furthermore, the rachitic condition of his spine indicates the nature of his trouble. Both corneæ are hazy, and at different points there are thick opacities, sufficient to obscure his vision, so that he is compelled to be lead about. Around the cornea, the circle of redness known as "ciliary injection," is clearly visible, and there are photophobia, lachrymation, etc., present. This circle of ciliary blood-vessels is *always* an indication of an inflammation, or at any rate, an irritation either of the cornea or of the iris, or of some of the deeper-lying structures of the ball. Characteristic of this scrofulous inflammation of the cornea, is the location or position of the deposit, viz: *in between* the layers of, (inter-lamellar) and not *upon* the cornea itself. Ordinary corneal inflammations are prone to suppuration, but scrofulous keratitis *never* results in the formation of pus, though it may have existed ever so long and severely. The disease lasts for several months, not unfrequently for more than a year, but, as a rule, it is ultimately curable.

We will put the patient upon cod-liver oil, iodide potassium and tonics, and direct him to take much outdoor exercise. Locally we will apply atropine as a sedative, and dust calomel upon the inside of the lower lid once daily, watching its effect from day to day. After the excessive irritability has passed away, we will direct to be placed upon inside of lower lids, every night at bedtime, a small piece of salve, composed of hyd. ox. flav. gr. ij., ung. aquæ rosæ ʒj. This is with the view of stimulating the vessels to renewed action, and to take up the deposits.

NOTE.—After being under this treatment for several months, the corneæ have gradually cleared up sufficiently to enable the boy to attend to his ordinary duties. Will continue the remedies, and hope to be able to dismiss him cured before many months.

Case XXVII.—*Irido-Choroiditis, with Exudative Membrane closing the Pupil—Iridectomy.* J. W., colored, æt. 56, Atlanta; had four years ago a severe inflammation (probably syphilitic) in the right eye, which resulted in the total destruction and collapse of the ball. About a year afterwards had sympathetic irido-choroiditis in the left eye, which ended in blindness, closing up the pupil with an exudative deposit, resulting from the inflammation. Usually an operation in such cases does no good, from the fact that the choroid has been so seriously impaired that restoration to vision is impossible. The perception of light, however, was so keen in the left eye that it was thought the choroid must not have suffered so severely, and hence an operation was determined upon.

A large artificial pupil was made by cutting away the upper portion of the iris, (iridectomy) and it was found that the exudation which filled the pupil also covered the entire lens in front, no portion being visible behind the new-formed pupil. The window, through which the light could stream into the eye, being enlarged, a greater volume of light could be discerned by the patient; but so long as this thick membrane covered the front of the lens, of course vision was impossible. To remove this membrane would necessitate the removal of the lens, and as this was not thought to be cataractous, and hence in a soft condition, it was not thought prudent to interfere further. The wound healed rapidly, and he was at his usual avocation (wood sawing) after a few days.

This case only serves to confirm my experience, that it is utterly useless to interfere (surgically or otherwise) with an eye that has gone through a sympathetic inflammation, with the view of restoring sight. It invariably results in no good. The perception of light may be tolerably good, and thus entice the surgeon to an operation, but always some such obstacle as above mentioned plants itself in the way to even a partial return to vision.

Case XXVIII.—*External Strabismus, with Paralysis of the Internal Rectus. Division of the External Muscle and Advancement (and thereby shortening) of the Internal.* C. H., colored girl, æt. nineteen, Georgia; had severe inflammation several years ago, which resulted in several small opacities of the cornea, and a partial paralysis of the internal rectus of the right eye, thus allowing the external muscle to draw the eye far outwards. After the division of the external muscle, it was found that the internal was not sufficiently powerful to bring the eye out of its abnormal position. The advancement operation was then made upon the internal rectus in the following manner: An incision was made through the conjunctiva, about two lines in rear of the edge of the cornea, over the insertion of the muscle, and a tenotomy hook passed entirely under it, bringing it fully into view. Three threads were then used (each armed with two needles) one being passed through the upper edge of the muscle, near its insertion; another through the lower edge, and the third through the middle. The needles of each thread were passed from the under surface of the muscle, so that the loop of each rested upon the sclerotic side. Next the conjunctiva was loosened from the sclerotic, from the point of the incision up to the edge of the cornea. The muscle was now severed from its insertion and freed from all its attachments, and the different threads passed in their regular order underneath the loosened conjunctiva and through the same, at its junction with the cornea. Upon being tightened and tied, the muscle was brought from its former insertion (about two lines behind the cornea) up to the very edge of the cornea, being covered at the same time by the conjunctiva. In other words, the muscle was shortened to this extent, and thereby the eye drawn very nearly into its proper position.

Comparatively little inflammation followed the operation, and after the third day the stitches were removed, the muscle having become firmly adherent at its new insertion. The eye turns a very little outwards still, but so slightly as not to be very noticeable. The internal muscle now acts very well, having very nearly recovered from the partial paralysis.

Case XXIX.—*Chalazion* (*torsal-cyst*) *and its Treatment.* H. G., Georgia, æt. twenty-seven, has a tumor of the size of a large pea upon the inside of the left lower lid. It is quite visible from the outside, and seems to be just under the skin, but upon pulling the lid down, it will be seen that it points towards the inner side of the lid. These tumors are known as chalazion, and are the result of an inflammation and a closure of one or more of the meibomian glands. At first the contents of the closed duct are soft, but after remaining for some time they become quite hard, and when let out present a cheesy appearance. The best way, and the quickest, of getting rid of such tumors is to evert the lid and open it freely, thus letting out the contents that have been for a greater or less time pent up. As often as they recur, or others form, they should be at once opened. Some astringent wash will often overcome the tendency to inflammation in the meibomian glands. When once formed and let alone, they not unfrequently remain for years as a small shot-like lump, which can be easily felt by passing the finger gently over the outer surface of the lid.

